# The VEGPREV study: effectiveness of four plant-based diets on weight loss, metabolic syndrome components and appetitive traits in overweight and obese individuals: a randomized controlled trial

**DOI:** 10.3389/fnut.2025.1677496

**Published:** 2025-11-18

**Authors:** Klaudia Wiśniewska, Katarzyna Małgorzata Okrȩglicka, Monika Paskudzka, Anna Maja Jagielska, Julia Bober, Michał Oczkowski, Olga Ciepiela, Aneta Nitsch-Osuch

**Affiliations:** 1 Department of Social Medicine and Public Health, Medical University of Warsaw, Warsaw, Poland; 2 Doctoral School, Medical University of Warsaw, Warsaw, Poland; 3 Central Laboratory, University Clinical Centre, Medical University of Warsaw, Warsaw, Poland; 4 Department of Laboratory Medicine, Medical University of Warsaw, Warsaw, Poland; 5 Department of Nutrition and Epidemiology, Medical University of Lodz, Lodz, Poland; 6 International Doctoral School, Medical University of Lodz, Lodz, Poland; 7 Department of Dietetics, Institute of Human Nutrition Sciences, Warsaw University of Life Sciences (WULS-SGGW), Warsaw, Poland

**Keywords:** plant-based diets, weight loss, metabolic syndrome, obesity, appetitive traits, dietary intervention

## Abstract

**Background:**

Obesity and its associated metabolic disturbances remain a growing public health concern, highlighting the need for effective and sustainable dietary strategies. This randomized controlled trial aimed to compare the effects of four plant-based dietary patterns: vegan (VG), lacto-ovo-vegetarian (LOV), Mediterranean (MD), and the EAT-Lancet Planetary Health Diet (EAT) with a control diet based on WHO recommendations (CTRL) in reducing body weight and improving selected metabolic and behavioral parameters in overweight and obese adults.

**Methods:**

A total of 90 participants (aged 18–64) were methodically assigned to one of five distinct dietary groups for a period of 12 weeks. The primary outcome measured was the change in body weight. Secondary outcomes encompassed body composition, waist circumference, energy and macronutrient intake, fasting plasma glucose and insulin levels, arterial blood pressure, plasma lipid profile, appetitive traits, physical activity, and quality of life. A total of 85 subjects completed the intervention.

**Results:**

All plant-based diets resulted in a reduction of body weight, waist circumference, fat mass, and energy intake in comparison to the control group. The most pronounced body weight reductions were observed after 12 weeks in the VG group (−6.7%) and the EAT group (−5.6%) (*p* < 0.001). A significant decrease in fat mass was observed across all intervention groups (*p* < 0.01). The investigation revealed no substantial between-group disparities in fasting plasma glucose, insulin, HOMA-IR, lipid levels, blood pressure or appetitive traits. No serious adverse events were observed.

**Conclusion:**

Among plant-based dietary patterns differing in animal product content, the EAT and VG diets demonstrated the most pronounced effects on weight and body composition. These findings provide support for the notion that structured, plant-based dietary interventions can be effective strategies for managing body weight.

## 1 Introduction

Obesity and related metabolic disorders have become a major public health challenge worldwide. The findings reported by the GBD 2021 Adult BMI Collaborators show that over 45% of the global adult population, including 1 billion males and 1.1 billion females, are affected by overweight or obesity (1). By 2035, these numbers are projected to rise to 3.3 billion, representing 54% of the adult population (2).

Obesity is characterized by an excessive accumulation of adipose tissue and is closely associated with hypertension (3), impaired carbohydrate (4) and lipid metabolism (5, 6). These issues have been linked to metabolic syndrome (MetS), which has been shown to contribute to the development of cardiovascular disease (CVD) and type 2 diabetes mellitus (T2DM) (7, 8). Consequently, a significant body of current research is focused on the development of optimal and effective treatments for obesity, as well as its potential complications (9).

Despite the pharmacological methods for obesity treatment, behavioral interventions, including dietary modification, continue to be regarded as both efficacious and the preferred treatment option (10–12). A notable solution for weight reduction that has garnered considerable interest is the adoption of plant-based diets (13–15). These diets are distinguished by their beneficial effects on individual components of the metabolic syndrome (16, 17). Additionally, they are gaining popularity among patients, largely due to their growing emphasis on environmental and ethical considerations, as evidenced by their inclusion in expert recommendations (18–20). The health benefits of various types of plant-based diets, such as vegan, lacto-ovo-vegetarian, or flexitarian, which involve the reduction or elimination of animal-based products, are of great interest (21–23). Nevertheless, there is a paucity of scientific evidence comparing the effectiveness of this type of intervention. Most of the available data comes from epidemiological studies. The health benefits of plant-based diets (PBDs) are primarily attributed to reduced calorie intake, although modifying some macronutrients, such as replacing animal-derived protein with plant-based protein, may also offer significant advantages in managing obesity and its related complications (24, 25). The relevance of understanding the links between PBDs and specific appetitive traits is also indicated, which may be crucial in the context of efforts to prevent the overweight and obesity epidemic. This assertion is supported by the findings of prior studies, which demonstrate a correlation between appetitive traits and body weight (26–28). On the other hand, research has shown that adopting PBDs, especially those with multiple restrictions like a vegan diet, can lead to various challenges (29). There is a need to identify a suitable dietary model that can effectively reduce body weight, optimize metabolic parameters, be environmentally sustainable, and be acceptable to patients. Therefore, it is reasonable to verify the efficacy of different types of plant-based diets in terms of weight reduction and improving the components of metabolic syndrome in individuals, compared to dietary patterns recommended by the World Health Organization.

In this study, a 12-week randomized controlled trial was conducted to compare the weight loss effects of plant-based diets: Vegan (VG), Lacto-ovo-vegetarian (LOV), EAT-Lancet Planetary Health Diet (EAT) and Mediterranean diets (MD) with traditional (CTRL – control) diet following the WHO recommendations. The study also aimed to determine which of the dietary models examined was most effective in improving metabolic risk factors in overweight individuals. The dietary patterns examined in this trial represented a spectrum of plant-based eating models differing in their inclusion of animal products. The Mediterranean diet is characterized by a high consumption of fruits, vegetables, whole grains, legumes, nuts, and olive oil, with moderate intake of fish and dairy products (30, 31). The EAT-Lancet Planetary Health Diet advocates a flexitarian approach, characterized by the consumption of predominantly plant-derived foods and limited amounts of animal products (18, 20). The lacto-ovo-vegetarian diet excludes meat, poultry, and fish but permits the consumption of eggs and dairy products. The vegan diet is entirely plant-based, eliminating all animal and animal-derived foods (32–34). The control diet was based on World Health Organization recommendations, emphasizing the daily consumption of fruit, vegetables, whole grains, and low-fat dairy products. Participants in this variant could also consume meat, fish, seafood, dairy products, and eggs (35). The application of this gradation of dietary models permitted the comparison of divergent levels of plant-based eating and their potential effects on body weight and metabolic outcomes.

The hypothesis of this study was that adopting PBDs would be associated with a significant reduction in body weight in obese subjects compared to the CTRL group, with the most pronounced effect observed in individuals from the VG group. It was further assumed that patients from the VG group would experience the greatest improvements in body composition, carbohydrate metabolism parameters (fasting plasma glucose and insulin concentration, HOMA-IR index), arterial blood pressure, lipid parameters, overall health status, and appetitive traits.

As part of this intervention, a comprehensive range of assessments was conducted to capture the multidimensional effects of dietary modification. The evaluations encompassed a range of domains, including biochemical, anthropometric, and behavioral aspects, along with sociodemographic and lifestyle factors. Standardized and validated tools were employed throughout the study to ensure data reliability. In addition to the present analysis, which focuses on anthropometric and metabolic outcomes, several other components of the VEGPREV project, such as inflammatory and cardiovascular biomarkers, liver health parameters, and psychosocial outcomes, are currently under development and will be presented in separate thematic publications.

The findings from this 12-week study are expected to support the refinement of dietary programs for individuals with obesity and metabolic disorders, and to inform the development of practical tools that enhance the effectiveness of weight-management strategies (29–32, 36).

The results of this 12-week study are expected to support the refinement of dietary programmes for individuals with obesity and metabolic disorders, and to inform the development of practical tools that enhance the effectiveness of weight-management strategies.

## 2 Materials and methods

### 2.1 Study design, participants and recruitment

A randomized, controlled trial (RCT) was conducted over a 12-week period, during which participants were randomly allocated in a 1:1:1:1:1 ratio to one of five groups: (1) MD, (2) EAT, (3) LOV, (4) VG, and (5) CTRL with WHO dietary recommendations. The randomisation schedule was developed and managed by an independent member of the research team, and the allocation was made using a computer-generated randomisation schedule.

The primary outcome measure was the mean difference in incremental weight change of 1% between each of the five groups [based on (23, 43)].

The secondary endpoints of the study included changes in energy and macronutrient intake, body mass, body composition, waist circumference, fasting plasma glucose and insulin levels, changes in HOMA-IR, blood pressure, blood lipid serum concentrations, and changes in appetitive traits, physical activity level, and quality of life indices.

The study was conducted in the Department of Social Medicine and Public Health at the Medical University of Warsaw, Poland, approved by the Local Bioethics Committee at the Warsaw Medical University (KB/37/2023) and conducted in accordance with the 1975 Declaration of Helsinki (37). The study was also registered on ClinicalTrials.gov (NCT06886490).

The participants' recruitment was conducted electronically through social media and website advertisements. Participants were recruited using a continuous recruitment strategy from November 2023 to April 2024. The study population comprised 90 patients aged between 18 and 60 years. All participants were omnivorous prior to the study enrolment and were randomly assigned to one of the study groups. Each participant was required to provide written informed consent to participate in the study (38).

The inclusion criteria were as follows: (1) age: 18–64 years old; (2) ability to attend regular meetings with the study team; (3) ability to give informed consent; (4) BMI >30 kg/m^2^ or BMI 25-29.9 kg/m^2^ with waist circumference in women ≥80 cm, in men ≥94 cm; (5) stable body weight, defined as a change of less than ±10% of the current body weight during the 6 months prior to the study.

The exclusion criteria comprised the following: (1) attempts to change dietary habits, including reduction of energy intake and elimination of meat and animal-based products in the past 6 months; (2) severe medical conditions, including diabetes mellitus, alcoholism, major and extensive surgeries, chronic kidney or liver disease, a history of myocardial infarction, unstable angina pectoris within the last 6 months, history of stroke within the last 6 months, or cancer within the last 5 years; (3) diagnosed insomnia; (4) eating disorders; (5) pregnancy or breastfeeding; (6) use of medications that cause significant weight loss or may affect metabolic parameters (e.g., GLP-1 receptor agonists, other anti-obesity agents, or antidiabetic drugs such as insulin, metformin, SGLT2 inhibitors, or DPP-4 inhibitors, as well as corticosteroids); (7) use of enteral or parenteral nutrition; (8) psychiatric conditions that prevent participation in the study or cooperation with the investigators; (9) lack of consent to participate in the study.

Participants were instructed to refrain from introducing any dietary supplements or discontinuing any existing supplements during the study. It is noteworthy that none of the participants used any dietary supplements other than vitamin B12, which was administered to all subjects in equal doses. Participants could be excluded from the study in the event of pregnancy, initiation or modification of pharmacological treatment affecting metabolism (e.g., antidiabetic, antidepressant, or corticosteroid therapy), or the development of any acute or chronic condition that could interfere with adherence to the nutritional protocol. Exclusion also applied in cases of declared non-compliance with the assigned dietary regimen or failure to attend mandatory study visits during which the required assessments were performed.

### 2.2 Assessments

#### 2.2.1 Anthropometric measurements

A calibrated digital scale with a height meter (C315.60/150.OW-1, RADWAG) was used to record body weight (without shoes and heavy clothing) with an accuracy of 0.1 kg. The height was measured with an accuracy of 0.1 cm. Body mass index (BMI) was calculated as body weight (kg) divided by the square of height (m^2^). The waist circumference was measured by trained research staff and recorded to the nearest 0.1 cm using a flexible steel tape measure at a point midway between the iliac crest and the lower costal margin (lower rib) in accordance with standard protocols. Body composition was quantified by bioelectrical impedance analysis (BIA) using a body fat analyser (Maltron BioScan 920) with an operating frequency of 50 kHz at 800 μA in the supine position. Tests were conducted at the outset of the study and at weeks 6 and 12.

#### 2.2.2 Biochemical measurements

The venous blood samples were collected into serum tubes with gel separator and centrifuged at 3,000 rpm at 20 °C for 10 min promptly after clot formation. Serum fasting glucose, insulin, and lipid profile were determined in the Central Laboratory, Central Clinical Hospital, University Clinical Center of the Medical University of Warsaw using the Cobas pro system (Roche Diagnostics). The LDL-cholesterol concentration was calculated using the Friedewald equation: LDL-C [mg/dL] = TC [mg/dL] – HDL-C [mg/dL] – TG [mg/dL]/5. For samples with triglycerides (TG) results exceeding 400 mg/dL, serum LDL-C was determined using the direct method. Non-HDL concentration was calculated as the difference between TC and LDL-cholesterol (mg/dl). The HOMA-IR index was calculated according to the equation: (serum fasting insulin concentration [IU/L] x serum fasting glucose concentration [mg/dl])/405.

Blood samples were collected at the commencement of the study, at week 6, and at the conclusion of the study. Fasting blood samples were collected every time in the morning (07:00–10:00) after an overnight fast of at least 8 h. The plasma, serum, and whole blood fractions were collected and analyzed immediately or stored at −80 °C until assayed.

#### 2.2.3 Blood pressure measurement

Patients' arterial blood pressure was measured in the office using an automatic oscillometric sphygmomanometer, using a cuff size appropriate to the arm circumference and following a standard procedure (39). All measurements were taken on the left arm after a period of at least 8 h without food, smoking or physical activity, and the patient remained seated for 5 min before starting the BP measurement. The cuff was placed at heart height, and three measurements were taken 2 min apart. The mean of the BP measurements was recorded at each visit.

#### 2.2.4 Dietary assessment and compliance

The participants were provided with comprehensive instructions regarding their dietary regimens. These dietary plans were discussed at each meeting with the dietitian and during weekly telephone consultations. The degree of compliance with the implementation of the recommendations and dietary patterns was evaluated through additional unannounced telephone calls, during which an interview was conducted to determine the subject's food and beverage consumption over the previous 24 h. On occasion, the subjects were also asked to submit photographic documentation of the meals they prepared. Furthermore, dietary intake was documented using a 24-h food record at the commencement of the study and at week 12. Prior to each of these assessments, participants received a relevant questionnaire and a set of written instructions from a participating dietitian on how to complete the log. Participants were asked to record all food and beverage intake using standard household utensils, such as measuring cups, spoons, and tablespoons. All food records were entered into the Diet 6.D computer program, which was designed for the purpose of planning and ongoing assessment of individual nutrition for the Polish population. The results of this assessment were then compared with the dietary standards for the Polish population, as defined in the Dietary Intake Standards for the Polish population (40).

Since the participants were informed about the type of diet they would follow, they were not blinded to their group assignment, nor were the other participants. To improve adherence to the dietary regimen at the beginning of the study, a thorough analysis was conducted to identify potential risks and strategies to mitigate them. All members of the research team then followed these established procedures, as detailed in Table 1.

**Table 1:** Analysis of potential risks in complying with the testing protocol.

**Potential risks**	**Solution method**
Resignation of participants from the study	A greater number of participants were recruited. Based on the literature review, we estimated a dropout rate of 30%.
Difficulties in implementing diet and nutrition recommendations	The materials for the patients were clear and concise. They have been tested in our previous study. The recipes were simple, using only commonly available and inexpensive products. Regardless of the type of diet.
Lack of motivation in following a diet	Based on the literature and our own long-term experience with obese patients, we have diversified the contact model with patients and intensified the contact frequencies (once a week video/call with an appropriately trained psychodietitian).
Worse wellbeing of patients during the diet	For patients who experienced changes in mood due to dietary changes, the research team made all efforts to ensure that the diet was well-balanced and properly composed. In addition, an internal medicine physician supervised the study.
The problem with compliance with the implementation of the recommendations and dietary patterns	Compliance with the implementation of the recommendations and dietary patterns were assessed through additional unannounced telephone calls in advance, during which an interview was conducted about the consumption of the last 24 h.
Effect of macronutrients on particular parameters	To minimize differences between groups and the potential impact of nutrients, the same assumptions were made for all dietary interventions.
Influence of other lifestyle elements on the analyzed parameter changes	Patients were advised not to make lifestyle changes, including physical activity, diet and use of stimulants outside the study design. In addition, questions about lifestyle changes were asked during check-up visits.
Omitting to consult and do examinations	Text messages with appointment reminders were sent.

#### 2.2.5 Questionnaire-based assessment

The assessment of the appetitive traits in the study participants was carried out using the Adult Eating Behavior Questionnaire (AEBQ) (26). The AEBQ measures eight appetitive traits: hunger (H), food responsiveness (FR), emotional over-eating (EOE), enjoyment of food (EF), satiety responsiveness (SR), emotional under-eating (EUE), food fussiness (FF), and slowness in eating (SE). The participants completed the questionnaire at the beginning of the study and again at the end of the final week.

Quality of life was evaluated using the SF-36 questionnaire (41). This tool is dedicated to subjectively assessing an individual's health status and consists of 11 questions and 36 statements that cover eight areas of functioning: physical fitness; limitations due to health problems; experience of pain; general health and wellbeing; energy level (vitality); social functioning; emotional state; and mental health. The quality of life index is calculated by summing the scores from each of the eight scales, providing a comprehensive assessment of overall health. In the Polish version of the questionnaire, a higher score indicates a lower quality of life, while a lower score indicates a higher quality of life. Participants completed the questionnaire at both the beginning and the end of the study.

During the study, participants were required to maintain their usual physical activity habits. They received detailed instructions to avoid any significant increases in physical activity and to refrain from introducing any additional exercise or training. It was essential for participants to report any changes in their physical activity to the research team. Physical activity data were collected using the International Physical Activity Questionnaire-Short Form (IPAQ-SF) (42). This questionnaire assessed physical activity over the past seven days and categorized it into four groups: (1) intense, (2) moderate, (3) walking, and (4) sedentary lifestyle. Additionally, the intensity, frequency, and duration of physical activity were evaluated. The data obtained from the IPAQ-SF was used to estimate the total amount of physical activity performed. This was achieved by weighting the reported minutes in each of the four categories of physical activity according to the estimated metabolic equivalent (MET) of energy expenditure. The total MET minutes *per* week were calculated by multiplying the MET duration (in minutes), frequency (in days), and intensity, then summing up the different categories of physical activity (vigorous, moderate, walking, and sedentary behavior). It was expected that participants would maintain their usual levels of physical activity throughout the intervention period. Physical activity levels were assessed at the beginning of the study and again at week 12.

### 2.3 Behavioral intervention

Patients enrolled in the 12-week study were allocated to a control group or one of the dietary intervention groups. They received dietary recommendations tailored to their assigned dietary patterns and were prescribed individualized meal plans. The primary dietary intervention focused on modifying the frequency and quantity of meat and meat-based product consumption, or their complete elimination, depending on the dietary model. Participant meetings with the dietitian were conducted mainly in person, with the option of remote follow-up sessions when necessary (hybrid format). The frequency of meetings remained consistent across all groups. To enhance motivation and support adherence to dietary recommendations, participants also received online consultations with a trained and experienced psychodietitian (Behavioral Nutrition Specialists). Patients were advised to refrain from making lifestyle modifications, including changes in physical activity, diet, and the use of stimulants, outside the parameters of the study design. The meetings were conducted according to a set schedule. During the dietary consultation, participants received dietary advice with a focus on practical aspects such as cooking, shopping, and reducing food waste. All necessary educational materials were also provided. An outline of the dietetic program for participants is presented in Table 2.

**Table 2:** Outline of dietary programme for intervention participants.

**Study week**	**Type of meeting**	**Description of the meeting**	**Received materials**
1	Face to face meeting	Dietary advice on how to compose meals, regularity of meal consumption and meal planning (cooking, shopping, reducing food waste). Providing the necessary information on the implementation of the dietetic plan.	Dietary plan Characteristics of the diet implemented in the diet plan
2	Online meeting	Question about the implementation of the diet plan.	Individual guidance on the implementation of the dietary plan
3	Online meeting	Conduct a review of the implementation of the dietary plan. Supporting the patient and motivating them to continue with the dietary plan.	Summary of the meeting
4	Online meeting	Question about the implementation of the diet plan.	Individual guidance on the implementation of the dietary plan
5	Online meeting	Question about the implementation of the diet plan.	Individual guidance on the implementation of the dietary plan
6	Face to face meeting	Conducting verification of the implementation of the dietary plan. Supporting the patient and motivating them to continue implementing the dietary plan. Provide the necessary information regarding the implementation of the new dietary plan with the new recipes.	Dietary plan
7	Online meeting	Question about the implementation of the diet plan.	Individual guidance on the implementation of the dietary plan
8	Online meeting	Question about the implementation of the diet plan.	Individual guidance on the implementation of the dietary plan
9	Online meeting	Conducting verification of the implementation of the dietary plan. Supporting the patient and motivating them to continue with the dietary plan.	Summary of the meeting
10	Online meeting	Question about the implementation of the diet plan.	Individual guidance on the implementation of the dietary plan
11	Online meeting	Question about the implementation of the diet plan.	Individual guidance on the implementation of the dietary plan
12	Face to face meeting	Conducting verification of the implementation of the dietary plan.	Summary of the dietary programme.

To reduce discrepancies between groups and mitigate the potential effects of nutrients, consistent assumptions were applied across or dietary interventions. An energy deficit of 15% of total energy expenditure was assumed. General assumptions for the dietary plans are provided in Table 3. The vitamin and major and trace elements for each dietary plan was aligned with the recommended daily intake based on the nutrition norms for the Polish population (40).

**Table 3:** General design framework and nutritional assumptions for all dietary interventions.

**Parameter**	**Assumptions**
Energy	85% of total daily energy expenditure
Protein	15-20% of the dietary energy
Fat	30-35% of dietary energy
Carbohydrates	50-55% of dietary energy

Values represent planning assumptions used by the research team to design the diet plans.

Patients were provided with specially designed diet plans that followed the general guidelines established in the dietitian's menu-planning program, which was based on the latest food databases. A key distinguishing feature of these diet plans was the content of animal and plant proteins, which influenced the frequency of consumption of various products, including meat, fish and seafood, dairy products, eggs, and their products (Table 4). Each diet included detailed instructions for meal preparation and a shopping list. The diets were tailored to the season, and the recipes were based on readily available products. Additionally, to ensure consistency across all groups and to prevent the potential adverse effects of nutrient deficiency, all participants received the same vitamin B12 supplement, which contained 100 μg of methylcobalamin per day. The supplement was provided in a convenient, easy-to-swallow chewable form, which was particularly useful for participants who had difficulty swallowing capsules. The product was suitable for vegans and vegetarians.

**Table 4:** Practical features of the dietary models: allowance of animal products, target protein ratio, daily core servings, fats, and exclusions.

**Type of diet**	**Animal products: allowance (per week)**	**Target animal: plant protein ratio**	Daily core servings^*^	**Dairy/alternatives (daily)**	**Fats: main sources**	**Key exclusions/notes**
CTRL	Red meat ≤ 3 servings ( ≤ 350 g cooked total); poultry 2–3; fish/seafood 1–2; eggs 3–7	≈80:20	Whole grains 4–6; legumes 1; vegetables 3–5; fruit 2–3; nuts/seeds 1	Dairy 1–3	Mixed oils, limit SFA; allow olive/rapeseed	Processed meat discouraged
MD	Red meat ≤ 1 small serving; poultry 1–2; fish/seafood 2–3; eggs 3-6	≈70:30	Whole grains 4–6; legumes 1–2; vegetables 4–6; fruit 2–3; nuts/seeds 1–2	Dairy 1–2 (yogurt/cheese preferred)	Extra-virgin olive oil primary; nuts; limit SFA	Processed meat minimized
EAT	Meat ≤ 100 g total/week; fish/invertebrates up to ~2 servings; eggs 2–4	≈50:50	Whole grains 4–6; legumes 2; vegetables 4–6; fruit 2–3; nuts/seeds 1–2	Dairy 1–2 (small portions) or fortified plant alternatives	Plant oils prioritized; limit SFA	Red/processed meat strongly limited
LOV	Meat/poultry/fish/seafood: 0; eggs 3–7	≈30:70	Whole grains 4–6; legumes 2; vegetables 4–6; fruit 2–3; nuts/seeds 1–2	Dairy 1–3 (or fortified alternatives)	Plant oils; nuts/seeds	Excludes meat, poultry, fish, seafood
VG	All animal products: 0	0:100	Whole grains 5–7; legumes 2–3; vegetables 4–6; fruit 2–3; nuts/seeds 1-2	Fortified plant alternatives 2–3 (soy/pea drinks, yogurts)	Plant oils; nuts/seeds	Excludes meat, poultry, fish, seafood, eggs, dairy, honey

^*^Standard serving definitions (for planning and reporting): whole grains = 1 serving = 40 g dry (e.g., oats, brown rice, whole-wheat pasta) or 1 slice whole-grain bread; legumes = 1 serving = 150 g cooked (or ~80 g tofu/tempeh); vegetables = 1 serving = 80 g; fruit = 1 serving = 150 g; nuts/seeds = 1 serving = 30 g; dairy = 1 serving = 250 ml milk/yogurt or 30 g cheese; eggs = 1 egg; fish/seafood = 1 serving = 100 g cooked; poultry/red meat = 1 serving = 100–120 g cooked. Ranges represent planning assumptions scaled to individual energy needs; macronutrient targets and the 15% energy deficit are specified in Table 3 (general framework). CTRL, control group; MD, Mediterranean diet; EAT, Eatwell diet; LOV, lacto-ovo-vegetarian diet; VG, vegan diet.

### 2.4 Statistical analysis

The study was powered to detect a significant difference in weight loss over a 12-week intervention period among five dietary groups, with an expected linear trend in percent body weight reduction from the the CTRL group, through the MD, EAT, LOV, to the VG. Following the methodology of Turner-McGrievy et al. (23) and Wright et al. (43), a mean incremental difference in percent body weight change of 1% was assumed successively between each of the five groups (corresponding to an effect size of 0.57). This 1% difference was used solely as a conservative statistical assumption for power calculation and was not intended to be a clinically meaningful threshold. Based on a pooled standard deviation (SD) of 2.5% and a significance level of α = 0.05, the minimum required sample size was estimated to be 60 participants, providing at least 80% power for detecting between-group differences of at least 2.4% in body weight for linear contrasts. To compensate for a potential dropout rate of up to 30%, the recruitment target was increased to 90 participants (18 per group). This total sample size was estimated to provide over 99% power to detect a significant linear trend in weight loss across the five dietary groups. The assumed linear order of the dietary groups (CTRL → MD → EAT → LOV → VG) was determined *a priori*, reflecting the progressive reduction in animal-derived food consumption across the models. This approach followed the analytical framework of Turner-McGrievy et al. (23), who applied a similar continuum from omnivorous to fully vegan diets to test for linear trends in anthropometric outcomes.

All statistical analyses were performed using IBM SPSS Statistics for Windows, Version 29.0 (IBM Corp., Armonk, NY, USA) and Statistica 13.3 version (TIBCO Software Inc., Palo Alto, CA, USA), depending on the type of analysis and data presentation. To assess the effect of diet type (MD, EAT, LOV, VG, and CTRL) over time (three measurements: baseline, at 6 weeks, and at 12 weeks) on body weight, waist circumference, body composition, lipid parameters, arterial blood pressure, carbohydrate metabolism (including serum fasting insulin and glucose levels and HOMA-IR index), appetite characteristics, level of physical activity, and quality of life, we performed a variance analysis with repeated measurement in a mixed scheme (5 groups × 3 measurements).

Before the main analysis, the assumption of sphericity of variance of covariance for intra-individual factors was verified using the Mauchly's test. In the cases where this test was not significant, and where only two measurement points were considered, the assumption of sphericity was adopted (44). If the Mauchly's test showed a significant violation of the sphericity assumption, the selection of the appropriate correction was based on the value of the ε coefficient (44). For the variables body weight, waist circumference, body fat percentage, LDL and HDL cholesterol levels, the Huynh–Feldt correction was used. For lean body mass and fasting insulin concentration, the Greenhouse–Geisser correction was employed. The differences between groups were analyzed by the Scheffe *post-hoc* test.

## 3 Results

### 3.1 Participant characteristics

**Figure 1:**
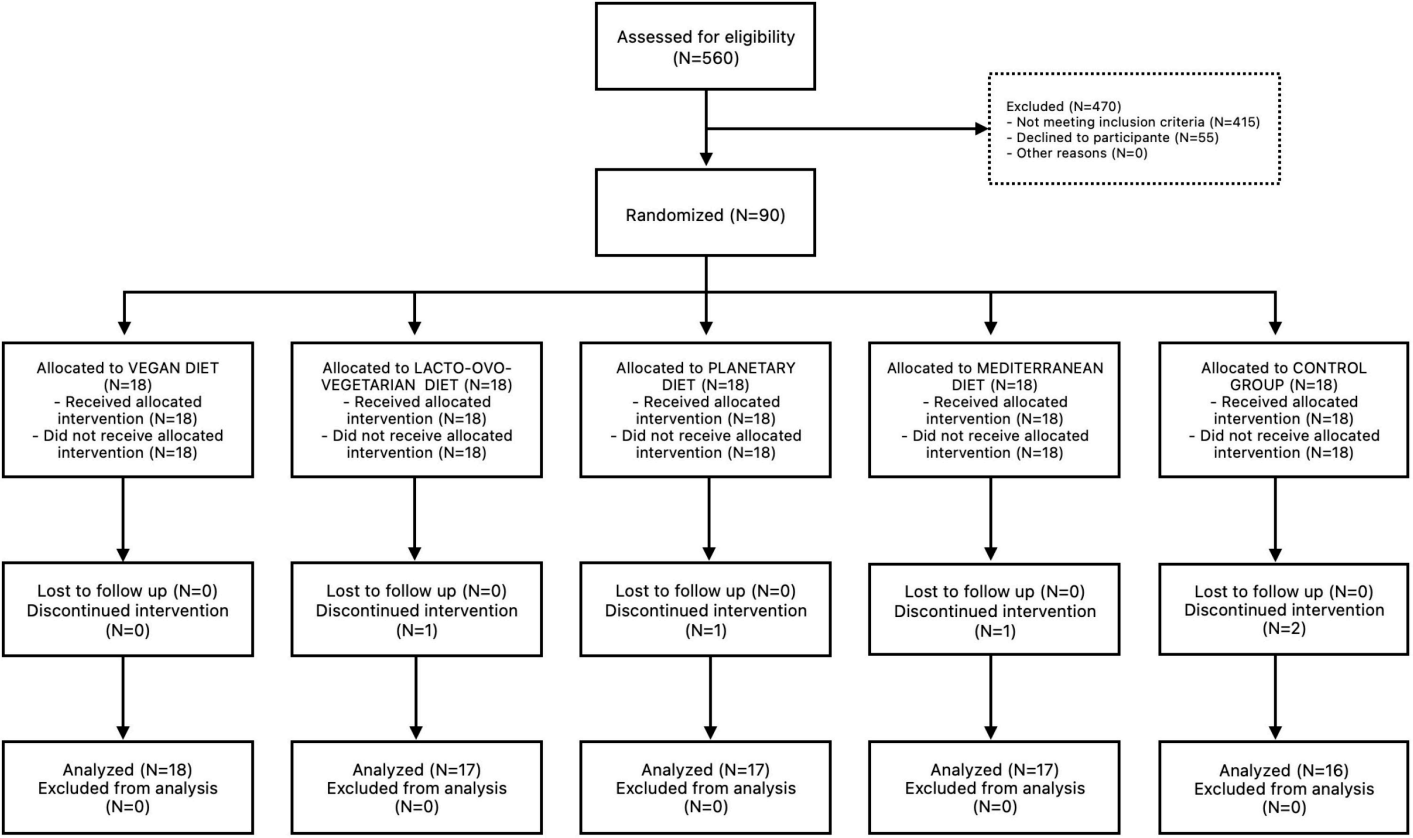
CONSORT Flow Diagram. A total of 560 participants were assessed for eligibility and 470 were excluded because they did not meet the inclusion criteria and 55 were excluded because they refused to participate in the study. Ninety participants were randomized to one of five groups: (1) the Mediterranean Diet (MD), (2) the EAT-Lancet Planetary Health Diet (EAT), (3) the Lacto-ovo-vegetarian Diet (LOV), (4) the Vegan Diet (VG), and (5) the control group (CTRL).

Ninety volunteers who met the eligibility criteria participated in the study. The 85 participants completed the 12-week intervention (94.4%). The full recruitment strategy is shown in the flowchart (Figure 1). Of the five participants who discontinued, two withdrew due to personal scheduling constraints, two were lost to follow-up, and one withdrew due to relocation. No serious adverse events were reported during the study.

The 85 participants were divided into five study groups [χ^2^(4, *N* = 85) = 0.12, *p* = 0.998; *N* = 16–18 subjects *per* group]. However, the gender distribution within the groups deviated significantly from randomisation [χ^2^(1, *N* = 85) = 14.41, *p* < 0.001], with approximately three-quarters of participants being female. However, the gender distribution within the subgroups was proportional [χ^2^(4, *N* = 85) = 1.36, *p* = 0.852, V = 0.127]. The gender and group breakdown of the sample is presented in Table 5. Descriptive statistics were calculated for three basic anthropometric indicators: height, body weight, and BMI, both for the entire sample and broken down by gender and inclusion into individual study groups. To determine the characteristics of the variable distributions, the range, mean, standard deviation (±SD), median, skewness, and kurtosis coefficients, as well as the values of the Shapiro–Wilk test (used to assess the normality of the distribution), were calculated.

**Table 5:** Gender and group breakdown in the study.

**Dietary intervention group**	**Total number of participants**		**Females**		**Males**	
	***N***	**%**	***N***	**%**	***N***	**%**
Mediterranean	17	20.0%	13	21.7	4	16.0
EAT-Lancet planetary health diet	17	20.0%	13	21.7	4	16.0
Lacto-ovo-vegetarian	17	20.0%	12	20.0	5	20.0
Vegan	18	21.2%	11	18.3	7	28.0
Control group	16	18.8%	11	18.3	5	20.0
Total	85	100.0%	60	70.6%	25	29.4%

In the analysis of the entire sample, most variables showed significant deviations from a normal distribution. The only exception was body weight, which followed a normal distribution. Additionally, the height data exhibited leptokurticity, indicating a strong concentration of results around the mean. When analyzing the data by gender, most variables displayed normal distribution, except for height in men and BMI in women, both of which showed statistically significant deviations from normality. For the men's height, the distribution was right-skewed, indicating a prevalence of lower values, and it was characterized by leptokurticity. Similar patterns emerged in the study groups: while most variables were normally distributed, the CTRL group showed a significant deviation in BMI, and the height of individuals in the EAT group also deviated from normal distribution. In the CTRL group, height demonstrated right-skewness and leptokurticity, suggesting a predominance of lower values and a concentration around the mean. Descriptive statistics for the quantitative indicators of demographic characteristics are detailed in Table 6.

**Table 6:** Descriptive statistics of indicators of quantitative demographic characteristics at baseline (*N* = 85).

**Parameter**	**Range**	**Mean**	**±SD**	**Median**	**Sk**	**Kurtosis**	**W**
**Total**							
Height [cm]	152.50–205.00	169.85	±9.23	168.50	0.74	1.21	0.96^*^
Weight [kg]	70.00–126.00	90.99	±11.51	90.00	0.53	0.33	0.98
BMI [kg/m^2^]	26.87–39.06	31.53	±3.09	30.86	0.47	−0.74	0.95^**^
**Female**							
Height [cm]	152.50–185.00	166.25	±6.95	165.25	0.41	−0.23	0.98
Weight [kg]	70.00–110.00	86.97	±9.29	87.50	0.36	−0.04	0.98
BMI [kg/m^2^]	26.98–39.06	31.51	±3.29	30.83	0.55	−0.81	0.93^**^
**Male**							
Height [cm]	166.00–205.00	178.50	±8.31	177.50	1.14	3.13	0.91^*^
Weight [kg]	79.00–126.00	100.62	±10.70	100.00	0.50	0.53	0.97
BMI [kg/m^2^]	26.87–36.75	31.58	±2.60	31.13	0.09	−0.81	0.98
**Mediterranean diet**							
Height [cm]	152.50–181.00	168.24	±8.45	168.00	−0.07	−1.03	0.95
Weight [kg]	70.00–108.30	93.84	±10.54	94.00	−0.66	0.02	0.94
BMI [kg/m^2^]	28.34–39.06	33.17	±3.16	32.49	0.27	−0.81	0.96
**EAT–Lancet planetary health diet**							
Height [cm]	154.50–185.00	171.18	±9.08	173.00	−0.40	−0.69	0.96
Weight [kg]	76.00–113.00	90.74	±10.32	91.00	0.66	0.41	0.94
BMI [kg/m^2^]	27.93–37.15	31.00	±3.04	29.87	0.94	−0.44	0.86^*^
**Lacto–ovo–vegetarian diet**							
Height [cm]	157.00–182.50	167.94	±7.46	167.50	0.38	−0.67	0.96
Weight [kg]	72.00–110.00	87.88	±10.52	84.00	0.97	0.47	0.90
BMI [kg/m^2^]	27.60–36.75	31.14	±2.81	30.83	0.42	−0.82	0.94
**Vegan diet**							
Height [cm]	156.00–189.50	171.39	±9.92	169.00	0.41	−1.03	0.94
Weight [kg]	70.00–122.00	90.83	±13.17	90.00	0.50	0.66	0.96
BMI [kg/m^2^]	26.87–35.43	30.85	±2.96	30.81	0.05	−1.42	0.92
**Control group**							
Height [cm]	157.00–205.00	170.47	±11.37	166.75	2.00	5.23	0.82^**^
Weight [kg]	74.50–126.00	91.70	±13.21	91.85	1.08	1.80	0.92
BMI [kg/m^2^]	27.73–38.19	31.54	±3.25	30.83	0.71	−0.49	0.92

^*^p < 0.05,^**^p < 0.01, SD, standard deviation; Sk, skewness; W, Shapiro–Wilk test.

### 3.2 Energy and macronutrient intake

Following 12 weeks of the study, reduced calorie intake was observed in all intervention groups (by 337.6 ± 229.2 kcal/day, 394.4 ± 243.6 kcal/day, 315.3 ± 221.4, and 554.9 ± 215.3 kcal/day for participants from MED, EAT, LOV and VG group, respectively) compared to CTRL diet (+77.5 ± 214.8 kcal kcal/day, *p* < 0.001). Mean daily protein intake (as % of energy intake) was also decreased in all intervention groups compared to CTRL one (15.5 ± 1.2, 15.1 ± 1.9, 15.0 ± 0.6 and 15.0 ± 0.6 *vs*. 18.0 ± 1.2, respectively; *p* < 0.001). At the same time, the mean daily fat intake (adjusted to % of energy intake) was reduced in the participants from MD (31.5 ± 3.8) *vs*. CTRL (34.5 ± 0.6; p < 0.05), LOV (34.2 ± 0.1; *p* < 0.05) and VG (34.6 ± 1.6; *p* < 0.01). Carbohydrates intake (expressed as % of daily energy intake) was increased in the participants from EAT compared to CTRL group (48.4 ± 2.4 vs. 43.8 ± 2.2; *p* < 0.01), however the intervention with MD, LOV and VG diets did not result in significant changes compared to CTRL diet (43.8 ± 3.4, 47.1 ± 2.0, and 46.8 ± 2.3, respectively). A comprehensive dataset illustrating these alterations is presented in Table 7.

**Table 7:** Changes in dietary intake.

**Variable**	**Time point**	**Dietary intervention**					***Post–hoc***
		**MED (A)**	**EAT (B)**	**LOV (C)**	**VEG (D)**	**CTRL (E)**	
Calorie intake [kcal/day]	I	2,406 (251)	2,373 (211)	2,283 (254)	2,429 (245)	2,353 (350)	II.A < I.A^***^, II.B < I.B^***^, II.C < I.C^***^, II.D < I.D^***^, IIB < II.E ^**^, IIC < II.E ^**^, IID < II.E ^***^
	III	2,068 (218)	1,979 (263)	1,968 (319)	1,874 (153)	2,430 (330)	
Proteins [% of daily energy]	I	14.8 (1.7)	15.1 (1.9)	15.1 (0.2)	14.5 (0.6)	17.5 (0.9)	I.A < I.E ^***^, I.B < I.E ^***^, I.C < I.E ^***^, I.D < I.E ^***^, II.A < II.E^***^, II.B < II.E^***^, II.C < II.E^***^, II.D < II.E^***^
	III	15.5 (1.2)	15.1 (2.0)	15.0 (0.6)	15.0 (0.6)	18.0 (1.2)	
Fats [% of daily energy]	I	31.5 (3.8)	32.8 (3.1)	34.2 (0.1)	34.6 (1.6)	34.5 (0.6)	n.s.
	III	33.2 (2.7)	32.2 (3.0)	34.0 (1.6)	34.1 (1.7)	34.8 (0.8)	
Carbohydrates [% of daily energy]	I	42.2 (5.2)	48.2 (1.7)	46.3 (0.1)	45.6 (2.4)	44.6 (1.5)	I.A < I.B ^***^, I.A < I.C^*^, II.A < IIB^*^
	III	43.9 (3.4)	48.4 (2.4)	47.1 (2.0)	46.8 (2.3)	43.8 (2.2)	

Data are expressed as means and standard deviations (SD). Time point: I, baseline; III, after 12 weeks of intervention; ^*^P < 0.05; ^**^P < 0.01; ^***^P < 0.001.

### 3.3 Summary of main and interaction effects

Analysis of the interaction effects between dietary regime and intervention duration revealed significant outcomes. Due to the relatively small size of the groups, *post-hoc* analyses included not only significance at the *p* < 0.05 level but also results showing a statistical trend (*p* < 0.10). The direction and magnitude of the observed differences are outlined in Table 8, while the precise data for all subgroups across measures are presented in Table 9. The following section provides a detailed description of interaction effects.

**Table 8:** Interaction effects for all analyzed variables: sphericity assessment, significance, and *post–hoc* tests.

**Variable**	**χ^2^(2)**	**ϵ**	***F***	***p***	**η^2^**	***Post–hoc***
Body weight [kg]	24.25^**^	0.791	12.13	< 0.001	0.183	II.A < I.A ^*^, III.A < I.A^**^, II.B < I.B^*^, III.B < I.B^**^, II.C < I.C^*^, III.C < I.C^**^, II.D < I.D^**^, III.D < I.D^**^
Waist circumference [cm]	18.10^**^	0.83	5.46	< 0.001	0.127	II.B < I.B^*^, II.D < I.D^*^, III.D < I.D^**^
Fat mass [%]	15.26^**^	0.851	4.02	< 0.001	0.108	III.A < I.A^**^, III.B < I.B^**^, III.D < I.D^**^
Fat–free mass [%]	128.78^**^	0.554	1.36	0.252	0.056	n.s.
Total cholesterol [mg/dL]	4.23	——	2.36	0.020	0.093	n.s.
LDL cholesterol [mg/dL]	9.10^*^	0.902	2.51	0.015	0.106	n.s.
HDL cholesterol [mg/dL]	6.75^*^	0.924	1.21	0.297	0.048	n.s.
non–HDL cholesterol [mg/dL]	5.46	——	2.34	0.021	0.098	n.s.
Triglycerides [mg/dL]	1.74	——	1.29	0.254	0.059	n.s.
Systolic blood pressure [mg/Hg]	4.52	——	0.48	0.872	0.021	n.s.
Diastolic blood pressure [mg/Hg]	4.05	——	0.48	0.868	0.022	n.s.
Fasting insulin	172.80^**^	0.530	0.86	0.490	0.041	n.s.
Glucose level [mg/dL]	0.4	——	1.08	0.381	0.049	n.s.
HOMA–IR	3.87	——	1.08	0.379	0.052	n.s.
Overall health status	——	——	1.46	0.224	0.059	n.s.
Total physical activity	——	——	1.41	0.237	0.065	n.s.
Food approach behavior	——	——	0.49	0.744	0.023	n.s.
Food avoidance behavior	——	——	0.94	0.444	0.045	n.s.

χ^2^ – Mauchly's variance sphericity test, ε – epsilon coefficient for the correction of the F test; Significance levels: ^*^p < 0.05, ^**^p < 0.01. I–III – subsequent measurement time points. A–E – study (dietary) groups; Scheffe's *post-hoc* test.

**Table 9:** Interaction effects for all analyzed variables: means, standard deviations, and *post–hoc* comparisons.

**Variable**	**Time point**	**Mediterranean diet (A)**	**EAT–Lancet planetary health diet (B)**	**Lacto–ovo– vegetarian diet (C)**	**Vegan diet (D)**	**Control group (E)**	***Post–hoc***
Body weight [kg]	I	94.85 (11.07)	91.06 (11.69)	88.61 (11.58)	91.16 (13.81)	92.82 (13.70)	II.A < I.A ^*^, III.A < I.A^**^, II.B < I.B^*^, III.B < I.B^**^, II.C < I.C^*^, III.C < I.C^**^, II.D < I.D^**^, III.D < I.D^**^
	II	92.19 (10.97)	88.43 (11.06)	85.86 (11.51)	87.39 (12.36)	92.44 (14.22)	
	III	90.62 (11.13)	85.89 (10.63)	84.64 (11.26)	84.98 (12.65)	93.36 (14.07)	
Waist circumference [cm]	I	103.26 (5.94)	100.15 (10.38)	102.35 (9.32)	103.78 (10.38)	99.81 (10.67)	II.B < I.B^*^, II.D < I.D^*^, III.D < I.D^**^
	II	100.41 (7.02)	98.32 (9.12)	99.82 (9.04)	99.58 (9.85)	97.84 (10.48)	
	III	99.59 (6.98)	94.35 (10.12)	96.88 (9.33)	96.50 (10.26)	99.31 (11.10)	
Fat mass [%]	I	42.54 (6.75)	39.47 (5.41)	39.47 (6.53)	37.36 (7.27)	39.51 (7.13)	III.A < I.A^**^, III.B < I.B^**^, III.D < I.D^**^
	II	41.19 (7.46)	38.72 (5.53)	38.61 (6.45)	36.12 (7.10)	38.92 (7.50)	
	III	40.53 (7.65)	37.50 (5.43)	37.85 (6.53)	35.15 (7.29)	39.55 (7.20)	
Fat–free mass [%]	I	57.47 (6.74)	57.74 (10.57)	60.53 (6.53)	62.64 (7.27)	60.49 (7.13)	n.s.
	II	58.81 (7.46)	61.28 (5.53)	61.39 (6.45)	63.89 (7.10)	61.09 (7.50)	
	III	59.48 (7.65)	61.85 (4.99)	62.15 (6.53)	64.85 (7.29)	60.45 (7.21)	
Serum total cholesterol level [mg/dL]	I	193.94 (50.62)	202.29 (38.41)	192.65 (31.82)	180.22 (32.77)	191.56 (42.68)	n.s.
	II	182.47 (54.33)	180.24 (34.55)	181.35 (28.34)	154.06 (26.34)	197.50 (45.78)	
	III	182.76 (45.83)	187.88 (34.27)	191.53 (28.16)	165.17 (26.31)	185.94 (34.38)	
Serum LDL cholesterol level [mg/dL]	I	118.65 (46.15)	121.00 (33.55)	114.00 (23.25)	101.83 (25.74)	115.69 (36.89)	n.s.
	II	112.18 (40.29)	114.47 (30.61)	114.12 (23.03)	92.39 (22.59)	114.00 (32.59)	
	III	115.06 (48.29)	108.94 (32.39)	104.76 (21.52)	82.17 (22.94)	124.50 (43.39)	
Serum HDL cholesterol level [mg/dL]	I	45.82 (10.32)	54.94 (12.75)	56.00 (10.29)	53.33 (14.90)	52.94 (13.16)	n.s.
	II	43.35 (9.64)	49.29 (8.81)	51.41 (10.95)	46.94 (11.57)	51.31 (8.60)	
	III	45.18 (11.35)	48.35 (14.55)	51.82 (10.92)	49.00 (11.73)	50.94 (9.70)	
Non–HDL cholesterol level [mg/dL]	I	148.12 (47.76)	147.35 (37.42)	136.71 (30.78)	126.89 (30.31)	138.63 (40.75)	n.s.
	II	139.12 (51.31)	130.88 (38.60)	129.35 (27.63)	107.11 (28.84)	146.19 (48.02)	
	III	137.59 (41.96)	136.59 (36.00)	139.88 (27.21)	116.17 (31.22)	135.00 (35.01)	
Serum triglycerides level [mg/dL]	I	146.53 (50.15)	131.35 (44.21)	113.94 (58.90)	125.56 (75.27)	107.69 (90.14)	n.s.
	II	120.59 (38.54)	110.41 (58.46)	123.82 (99.80)	125.11 (75.63)	107.25 (72.61)	
	III	129.88 (50.17)	110.35 (43.07)	129.24 (60.00)	118.28 (71.82)	106.25 (64.60)	
Systolic blood pressure [mg/Hg]	I	136.47 (21.18)	137.82 (16.25)	137.76 (7.71)	133.28 (10.68)	128.06 (9.53)	n.s.
	II	133.71 (17.17)	132.59 (14.91)	135.65 (10.32)	128.28 (9.30)	124.81 (9.99)	
	III	132.94 (13.33)	128.94 (18.78)	134.88 (12.35)	126.22 (11.89)	125.75 (12.60)	
Diastolic blood pressure [mg/Hg]	I	85.06 (11.24)	86.18 (9.19)	84.71 (7.90)	81.89 (7.73)	78.13 (7.18)	n.s.
	II	82.65 (10.67)	82.71 (7.47)	85.59 (9.10)	79.94 (10.52)	78.06 (7.72)	
	III	82.18 (9.07)	82.76 (10.17)	83.06 (8.18)	79.56 (8.33)	76.25 (9.53)	
Serum fasting insulin level	I	14.43 (7.72)	9.92 (3.85)	11.13 (5.59)	10.54 (4.99)	10.72 (5.43)	n.s.
	II	12.31 (4.62)	10.23 (6.39)	9.73 (3.82)	9.00 (4.80)	10.50 (5.22)	
	III	14.50 (7.52)	10.96 (6.86)	21.24 (46.92)	9.44 (4.16)	10.43 (4.15)	
Serum glucose level [mg/dL]	I	90.18 (10.70)	88.35 (7.28)	90.47 (8.37)	89.56 (7.91)	86.81 (8.68)	n.s.
	II	89.71 (6.18)	89.82 (7.09)	90.88 (6.81)	89.56 (6.63)	91.25 (10.10)	
	III	93.12 (8.34)	89.65 (7.66)	91.65 (9.27)	89.72 (7.45)	90.94 (8.33)	
HOMA–IR	I	3.38 (1.87)	2.18 (0.90)	2.51 (1.40)	2.38 (1.27)	2.27 (1.20)	n.s.
	II	2.71 (0.96)	2.34 (1.55)	2.19 (0.93)	2.09 (1.10)	2.39 (1.39)	
	III	3.32 (1.76)	2.51 (1.74)	2.39 (1.11)	2.18 (0.99)	2.37 (1.07)	
Overall health status	I	117.53 (20.58)	122.31 (23.99)	130.19 (19.59)	122.50 (14.65)	106.80 (33.92)	n.s.
	II	——	——	——	——	——	
	III	126.47 (25.44)	132.37 (25.52)	139.50 (11.95)	123.61 (21.17)	109.73 (32.78)	
Total physical activity	I	738.98 (820.05)	1,062.01 (1,138.56)	1,234.00 (2,265.72)	753.39 (643.82)	700.50 (507.08)	n.s.
	II	——	——	——	——	——	
	III	1,079.06 (1,028.69)	883.12 (817.97)	1,150.38 (955.32)	1,391.56 (1,627.69)	687.69 (507.67)	
Food approach behavior	I	3.18 (0.74)	3.44 (0.67)	3.18 (0.57)	3.31 (0.68)	3.26 (0.72)	n.s.
	II	——	——	——	——	——	
	III	3.18 (0.65)	3.31 (0.78)	2.99 (0.74)	3.23 (0.67)	3.25 (0.77)	
Food avoidance behavior	I	2.21 (0.46)	2.14 (0.42)	2.07 (0.45)	1.99 (0.45)	1.95 (0.40)	n.s.
	II	——	——	——	——	——	
	III	2.33 (0.48)	2.21 (0.47)	2.18 (0.45)	2.12 (0.55)	2.06 (0.49)	

Time point: I, baseline; II, after 6 weeks; III, after 12 weeks of intervention; Scheffe's *post-hoc* significance: ^*^p < 0.05, ^**^p < 0.01.

#### 3.3.1 Changes in body weight and body composition

**Figure 2:**
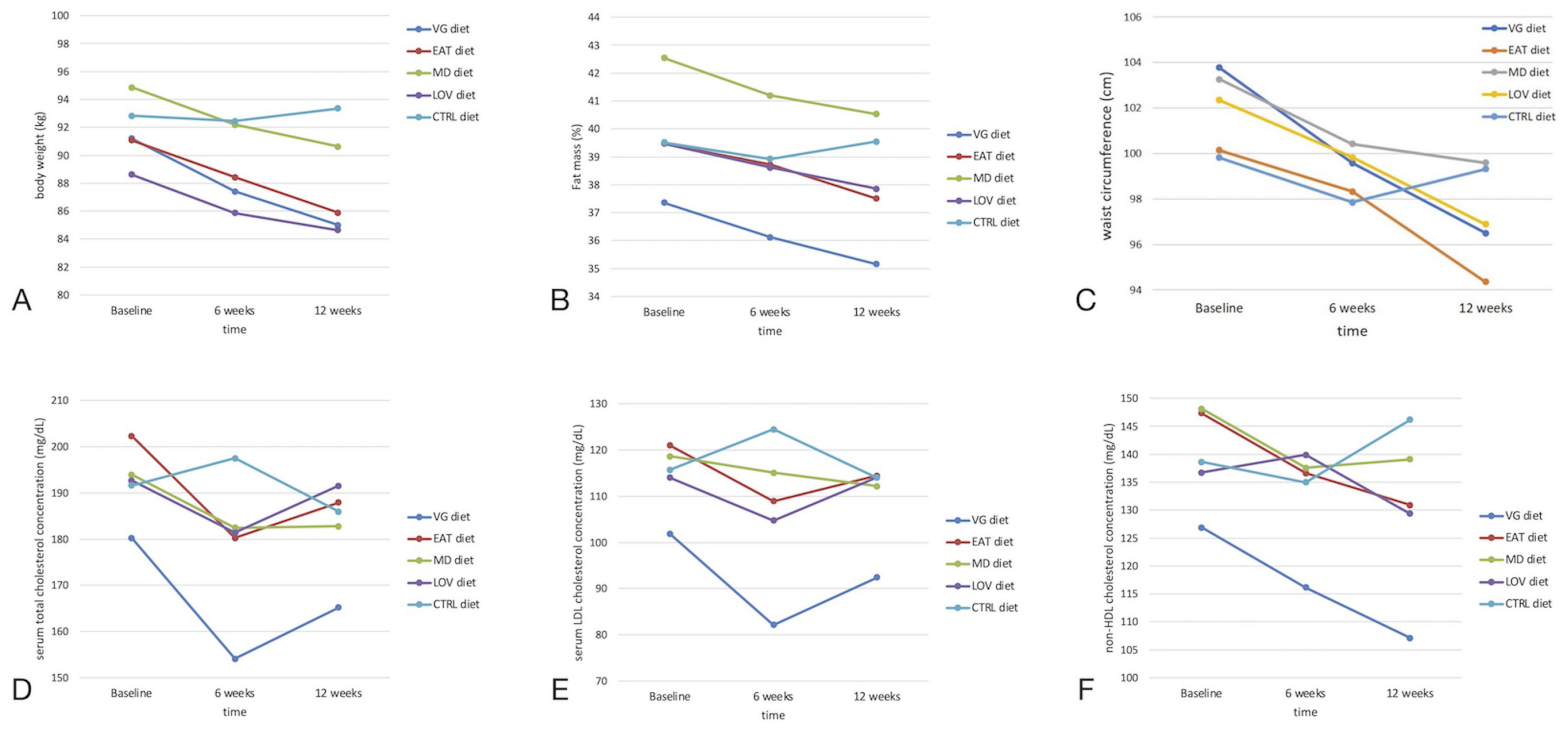
Changes in anthropometric and lipid-related parameters over time depending on the type of diet (VG, LOV, MD, EAT, CTRL). **(A)** Body weight (kg), **(B)** Fat mass (%), **(C)** Waist circumference (cm), **(D)** Total cholesterol (mg/dL), **(E)** LDL cholesterol (mg/dL), **(F)** Non-HDL cholesterol (mg/dL). Statistically significant effects of the diet × time interaction were observed for body weight, fat mass percentage, and waist circumference **(A–C)** (*p* < 0.05; repeated-measures ANOVA). No significant differences were observed for lipid parameters **(D–F)**. Data are presented as means ± SD. CTRL, control group.

The initial effect of the interaction pertains to body weight. In the experimental groups, a significant decrease in body weight was observed by week 6 and continued through week 12. In the CTRL group, body weight remained stable. The largest reductions were observed in the EAT and LOV groups, although these changes remained consistent with the direction and proportion of changes seen in the other groups. It is also worth noting that the study groups differed in the baseline level of body weight, which significantly affects the interpretation of the final values (Figure 2).

**Figure 3:**
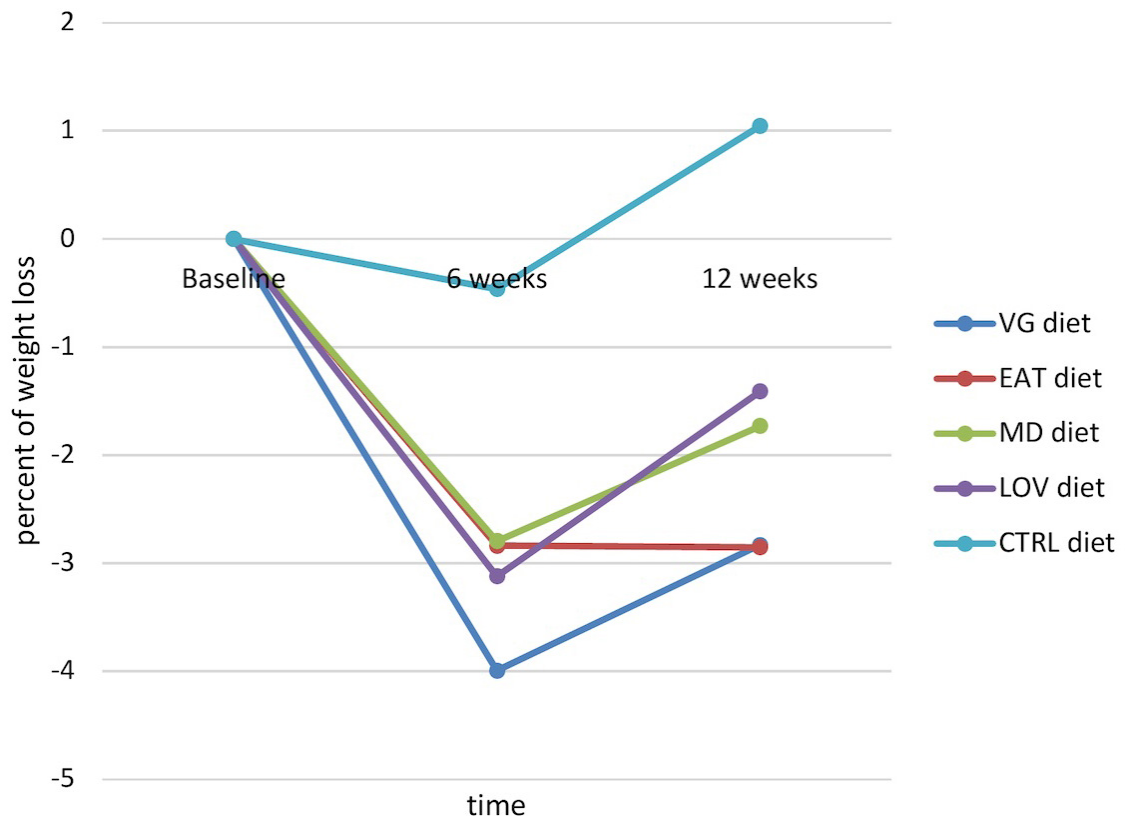
Dynamic in body weight changes expressed as a percentage of weight loss depending on the type of diet (significant effect of the interaction diet × time).

As illustrated in Figure 3, the most significant weight reduction was observed in the VG group (approximately −4%) up to week 6, with the EAT group exhibiting a loss of approximately −2.8%. In the LOV and MD groups, the decreases were about −3.1% and −2.8%, respectively. After 12 weeks, there was a partial decrease in body weight in all intervention groups, ranging from−1.4% in the LOV group to −2.9% in the EAT group, compared to the midpoint of the intervention. In the CTRL group, body weight remained unchanged. It remained within the range of ±1% throughout the entire period.

A gradual (linear) decrease in body fat levels from baseline to the end of the study was observed in all intervention groups, with a slightly larger reduction noted for the EAT (-1.97%) and MD diets (-2.0%). In contrast, the values in the CTRL group remained relatively stable (Figure 2).

#### 3.3.2 Effects on metabolic syndrome components: waist circumference, blood pressure, glucose metabolism, and lipid profile

Similar to body weight, there was a significant decrease in waist circumference (Figure 2) between the baseline measurement and the week 6 measurement in the experimental groups, particularly among those following the EAT (100.1 ± 10.4 cm vs. 98.3 ± 9.1 cm) and the LOV diet (102.3 ± 9.3 cm *vs*. 99.8 ± 9 cm). However, by week 12, there was a significant decrease in waist circumference compared to the baseline in the EAT (100.1 ± 10.4 vs. 94.3 ± 10.1; *p* < 0.001) and LOV group (from 102.3 ± 9.3 cm vs. 99.8 ± 9.0 cm; *p* < 0.001). In the CTRL group, the changes were minimal, although a slight decrease was observed throughout the entire follow-up period. Despite some differences in the dynamics of change at week 6, the decrease in waist circumference (between the beginning and the end of the study) ranged from - 7.3 ± 4.7 cm in the VG group to +0.5 ± 3.7 cm in the CTRL group.

Changes in the serum lipid profile were observed among participants in the intervention groups. The VG group exhibited the most pronounced, but non-significant, decrease in total cholesterol and LDL cholesterol (Figure 2), with concentrations gradually decreasing throughout the intervention period, reaching a low at week 12. There was also a moderate, systematic decline in the EAT group. In the LOV and MD groups, the serum LDL cholesterol concentration decreased by week 6, after which they stabilized or increased slightly. In the CTRL group, on the other hand, there was an opposite trend: after a slight drop by week 6, the LDL cholesterol concentration rose clearly above the baseline value in week 12. A similar pattern was observed for non-HDL cholesterol concentration in the VG group (Figure 2), with values decreasing steadily from baseline to week 12. In the EAT group, a gradual decline was also noted, although less pronounced. The subjects from the MD and LOV groups experienced a reduction in total cholesterol concentration mainly during the first phase of the study, after which their levels stabilized. The CTRL group exhibited the opposite trend: after a slight decrease by week 6, there was an increase that exceeded the baseline value by week 12. There were no statistically significant differences in serum triglyceride concentration between the subjects from CTRL and intervention groups after the dietary intervention. The parameters of carbohydrate metabolism, including serum fasting glucose and insulin concentration, as well as the HOMA-IR index, showed no substantial alterations in response to the dietary interventions compared to the CTRL group. A thorough examination of the findings revealed no statistically significant differences in systolic and diastolic blood pressure values between the CTRL and study groups (Table 8).

### 3.4 Changes in appetitive traits, physical activity, and quality of life

The assessment of appetitive traits in the study participants was carried out using the Adult Eating Behavior Questionnaire (AEBQ) (26), which had previously been evaluated in a separate study conducted on a cohort adhering to a plant-based diet (45). In the present study, AEBQ was administered at baseline (week 1) and after 12 weeks of dietary intervention. The repeated measures analysis (Table 8) and descriptive statistics (Table 9) showed no statistically significant effects of time, group, or their interaction for food approach and food avoidance traits. These findings indicate that eating behavior patterns remained stable across all dietary groups during the intervention period. Similarly, no significant differences were observed in physical activity levels or quality of life measures (Table 8).

## 4 Discussion

The present study demonstrated that a 12-week dietary intervention based on various plant-based eating patterns can lead to beneficial changes in body weight, waist circumference, and body composition, particularly among participants following the VG and EAT diets, who experienced the most consistent and pronounced reductions in these parameters. Although these changes did not reach statistical significance, they indicated a positive trend in plasma total cholesterol, LDL, and non-HDL cholesterol concentration, particularly in the VG and EAT groups compared to CTRL group. Analogous trends were observed in the other intervention groups; however, these changes were less pronounced or stabilized later in the study. With regard to other metabolic parameters, including plasma fasting glucose and insulin levels, the HOMA-IR index, and arterial blood pressure, no significant differences were observed between the intervention and control groups. Conversely, no substantial alterations in appetitive traits, physical activity levels, or quality of life were observed among the study participants in response to the dietary interventions. It is important to note that this study was the first to compare four different plant-based diets, each with varying levels of animal-based foods, against a control group consuming a control diet. The present comparison focuses not only on discrepancies in weight loss but also on the efficacy of the models in ameliorating specific elements of metabolic syndrome, appetitive traits, physical activity, and quality of life.

The health benefits of weight reduction are extensive. Research has shown that weight reduction is linked to improvements in many components of the metabolic syndrome (46, 47), including cases where this is achieved through a plant-based diet (16). The present study aimed to determine whether participants in all intervention groups would experience weight loss. The results indicated that this was the case, with the rate and magnitude of weight loss varying depending on the type of diet used and the time of measurement. By week 6, the most significant body weight reduction was observed in the VG group (approximately −4%), which may have been related to the most substantial decrease in energy intake in this group (average −555 kcal/day). Minor discrepancies were also observed between the planned and reported macronutrient composition, reflecting the usual variability in dietary adherence. Nevertheless, the intended nutritional structure and overall macronutrient balance of all intervention diets were maintained. This finding aligns with the observations reported by Turner-McGrievy et al. (23), who also documented substantial weight and calorie loss among individuals adhering to a vegan diet.

After a 12-week period, the subjects who had followed the EAT and VG diets achieved the lowest body weight, thus demonstrating their effectiveness throughout the intervention period. This result is consistent with the findings of previous observational studies (48–50) and the systematic review by Mambrini et al. (51) which identified the EAT-Lancet Planetary Health Diet as a promising tool for weight loss and obesity prevention. The LOV and MD groups experienced moderate weight loss, with a percentage change of approximately −3% and −2.8%, respectively, by week 6. This was followed by a further slight decrease or stabilization by week 12. No significant changes were observed in the CTRL group, as participants' body weights remained within ±1% throughout the study. It is also noteworthy that the participants did not receive financial compensation for their involvement in the study or reimbursement for travel expenses, which may indicate a high level of intrinsic motivation. Although minor baseline differences in body weight were observed between groups, these were statistically controlled for through the repeated measures design, which models changes within subjects over time.

A gradual (linear) decrease in body fat levels was observed from baseline to the conclusion of the study across all intervention groups. Notably, a slightly greater reduction was seen in participants from the EAT and MD group. This decrease was consistent at all measurement points, highlighting the importance of body composition analysis for these dietary interventions. Relying solely on body weight or BMI for dietary management may not provide an accurate picture. The improved outcomes for the EAT and MD diets may be attributable to a slightly higher protein content in these diets compared to the vegan diet, which could enhance feelings of satiety (50, 52, 53). It is important to note that the reductions in weight or body fat observed in the present study were smaller than in the study conducted by Kahleova and her team (54, 55). This may be due, in part, to differences in motivation, as participants in our study exhibited a lower baseline body mass index (31.53 ± 3.09). As a result, the rate of weight loss may have been reduced.

Waist circumference measurement is an established criterion for diagnosing metabolic syndrome; therefore, it was included in the current study. All experimental groups showed a noticeable decrease in waist circumference between the baseline measurement and the measurement at weeks 6 and 12. The effect was particularly significant for the EAT and VG diets. However, it is worth noting that this outcome is not universally observed. A meta-analysis of randomized trials by Melgar et al. (56) found that vegetarian diets were associated with body weight loss but did not result in a significant reduction in waist circumference (mean difference of −3.00 cm, 95% CI: −6.20 to 0.20).

Contrary to the findings of preceding studies, this intervention did not result in statistically significant changes in blood lipid concentrations, including plasma total cholesterol, LDL-C, non-HDL, and triglycerides, among the intervention groups compared to the CTRL group. Despite the presence of downward trends in total cholesterol and LDL-C levels within the VG and EAT-Lancet groups, these trends did not attain statistical significance. The absence of substantial variations in the results may be partially attributed to the low baseline lipid levels of the study participants and the moderate duration of the intervention. It is also conceivable that the effects of PBDs on the lipid profile are more pronounced in populations with dyslipidaemia, morbid obesity, or under strictly controlled conditions. Previous interventions, particularly those involving a low-fat vegan diet, have demonstrated favorable changes in TC and LDL-C levels (43, 57, 58). However, it should be noted that not all studies support these findings. For instance, a study comparing a low-fat vegan diet with the American Diabetes Association (ADA)-recommended diet in patients with type 2 diabetes found no significant differences in lipid profile between the groups (59, 60). A meta-analysis by Koch et al. (61) indicates that, although plant-based diets are associated with reductions in total cholesterol and LDL-C, these effects are most pronounced in studies with precise dietary control and high participant compliance. In the present study, participants exhibited a decline in body weight and body fat; however, the alterations in their lipid profile were not pronounced enough to attain statistical significance. This suggests that the impact of a plant-based diet on lipids may be contingent on the clinical context, the nature of the diet, and the duration and intensity of the intervention.

The present study revealed no significant impact of dietary interventions on serum fasting glucose and insulin concentration, as well as on HOMA-IR. It was observed that mean baseline glucose levels were within the normal range across all study groups, a factor that may have influenced the ultimate outcomes. A systematic review and meta-analysis of randomized controlled trials (62) examined the effect of plant-based diets on markers of insulin sensitivity. In comparison with control diets, plant-based diets have been shown to improve HOMA-IR (95% CI: −1.67 to −0.27; *p* = 0.007) and fasting serum insulin concentration (95% CI: −7.22 to −1.04; *p* = 0.009) in overweight/obese subjects. The findings from a parallel meta-analysis (63) confirmed that vegetarian diets significantly reduced fasting serum blood glucose and glycated hemoglobin concentration in comparison with omnivore diets, especially when the period of the diet was longer than 12 weeks. The impact was especially pronounced in the vegan diet.

There has been a limited number of studies on the effects of PBDs on the changes in arterial blood pressure. In this study, no significant changes in systolic or diastolic blood pressure were observed. The baseline blood pressure levels of the subjects may be a contributing factor to the observed variations in outcomes. The duration of the intervention, which was insufficient, may also be an insufficient factor. In the present study, mean baseline blood pressure levels were found to be close to normal values. The (63) study by Mishra et al. (58) on the effects of vegetarian and vegan diets on systolic and diastolic blood pressure found no significant differences. Similarly, in a review of RCTs analyzed by Melgar et al. (56) involving nine studies and a total of 1,628 overweight or obese participants, no significant effect of vegetarian and vegan diets on blood pressure was observed in comparison to conventional diets.

In the present study, the data regarding changes in individual appetitive traits resulting from the type of intervention are inherently organic. A comparable questionnaire was used in the study conducted by Gillies et al. (64), which also involved plant-based diet interventions. The TFEQ-R18 questionnaire was utilized to select participants, with the objective of excluding those at risk of eating disorders. Furthermore, the TFEQ was employed as a control variable (covariation) in the analysis of the effect of diet on adherence and subjective eating experiences. However, the TFEQ was not analyzed in relation to changes before and after the intervention, i.e., the impact of the diet on appetitive traits was not assessed. Our previous cross-sectional study demonstrated the potential of plant-based diets for beneficial changes in selected appetitive traits (45). For instance, food avoidance characteristics, such as food fussiness and slowness in eating, are clearly higher in the group following a plant-based diet. However, we have had no experience with the use of this questionnaire in randomized clinical trials to date. We suspect that a longer participant follow-up time may be required to observe changes in food avoidance and attitudes toward food.

No substantial changes were noted in the quality of life domain among the participants of the present study. We believe this is an important aspect, often overlooked, that should be assessed in dietary intervention studies. The quality of life of the participants under the influence of the diets, according to the studies, can be subject to alteration. In this specific case study, no decline in quality of life was observed during the 12-week period of even the more restrictive PBDs.

Physical activity was assessed using the validated IPAQ-SF questionnaire, a standard tool in dietary intervention research (65, 66). Although more advanced monitoring methods exist, the use of this validated and widely accessible instrument was considered appropriate given the study's design and focus on dietary intervention. No significant changes in physical activity levels were observed at any measurement point, nor between the study groups, suggesting that the differences observed in body weight and composition were attributable to dietary modification rather than changes in physical activity.

The present study faced several limitations. As is common with other dietary interventions, the participants in this study were not blinded to the intervention, despite the randomized trial. It is believed that knowing the type of diet used may influence more conscious behavioral changes in other areas of life (67, 68). Furthermore, the pool of participants for this study may have already shown interest in plant-based diets or a desire to improve their health through dietary modification. Additionally, the majority of the participants were female, a demographic that is often characterized by higher health consciousness and a greater engagement with health and nutrition subjects (69). This could partially explain the positive outcomes observed in the EAT-Lancet Planetary Health Diet. This dietary pattern was not as well-known to the study participants as the WHO recommendations or the vegan diet, and therefore aroused more curiosity, which could have potentially influenced the participants' motivation.

Adherence to dietary guidelines is a significant concern. We did not have full control over the study participants. Despite the implementation of a comprehensive protocol, regular meetings, and unannounced consultations, it is possible that participants have consumed supplementary products not included in their prescribed diet plans. Moreover, the long-term maintenance of a plant-based diet may present certain challenges for some participants, which may be attributable to factors such as taste preferences, product availability, cost, or established eating habits. This is partly illustrated by the increase in weight at the third measurement point after 12 weeks. Despite participants' assertions that they adhered to the stipulated protocols and consumed nutritionally adequate foodstuffs, even within the recommended products for the dietary model, it is conceivable that their energy intake may have exceeded the recommended levels and that they may have modified the diet plans to align with their personal preferences. This may be attributable to fatigue resulting from dietary adherence. This phenomenon was partially elucidated by Kolbuszewska et al. (70) in their study. This is due to the fact that they demonstrated that the degree of adherence to a diet is contingent on motivation. A plant-based diet, initiated primarily for health-related reasons, is more prone to a swift decline in motivation, often resulting in its premature cessation. Conversely, motivations of a moral or environmental nature exhibit greater persistence and serve as a significant catalyst for long-term adherence to plant-based diets. In this study, the significant factor of financial motivation was excluded, and participants were instead asked to provide information on any additional motivations. However, data on the relationship between motivation and other parameters is not yet available. Moreover, the enhanced energy value of the diet, while preserving its quality and adhering to the principles of the respective dietary models, may offer a partial explanation for the observed increase in body weight, accompanied by a concomitant enhancement in body composition, including a reduction in body fat. Furthermore, it is challenging to monitor spontaneous changes in lifestyle outside the diet. An attempt was made to verify this using the physical activity questionnaire; however, as with other questionnaires, the data are declarative and may be subject to bias.

In addition to limitations related to compliance with recommendations and lifestyle control, another important issue is the accuracy of dietary composition assessment. The micronutrient composition of the subjects' diets was not comprehensively assessed. Despite calculating the energy value and macronutrient distribution (i.e., protein, fat, and carbohydrate content) in all dietary plans, it was not possible to accurately assess micronutrient intake due to incomplete data on the content of certain nutrients in the foods consumed and reported by participants in their 24-h dietary diaries. Consequently, it was not feasible to quantify discrepancies in the intake of major and trace elements and vitamins, including zinc, magnesium, chromium, and vitamin C, which have been demonstrated to impact carbohydrate and lipid metabolism (71–74). Furthermore, the subjects' dietary intake was assessed using two 24-h dietary interviews – one at the commencement of the study and one after 12 weeks of intervention. Despite the fact that this approach provides only a snapshot of food intake at each time point, it remains a widely used and practical method in the field of dietary intervention studies. The interviews were collected by trained dietitians and used to estimate the overall direction and magnitude of changes in diet between the start and end of the intervention. Furthermore, no detailed assessment of individual nutrient classes, such as dietary fiber, fatty acid profiles and individual carbohydrate fractions, was conducted. This limitation should be taken into account when interpreting the study's metabolic results. Consequently, the precision of the habitual intake estimate in each dietary group is limited. In the interest of enhancing the comprehensiveness of the assessment, it would be advantageous to incorporate these parameters in subsequent studies.

It is also important to note that the study had a relatively small number of participants, which may not have been representative of the wider population. Moreover, the relatively short study duration of 12 weeks may not have been sufficient to assess additional parameters beyond body weight, selected body composition components, and lipid profile, as evidenced by the lack of statistical significance for blood pressure and carbohydrate metabolism parameters. Despite the prevalence of 12-week dietary interventions in research (21, 75). It is recommended that the recruitment of participants be expanded and the duration of the study be prolonged to facilitate the observation of additional changes. The long-term adherence to different plant-based diet models may present a significant challenge, particularly for individuals who have not previously restricted their meat intake.

Despite these difficulties, this study offers numerous advantages. This is the first study to compare five different dietary models with varying proportions of zoonotic products in terms of differences in weight loss, body composition, and efficacy in improving individual components of the metabolic syndrome and appetitive traits. A key distinction of this study was the recruitment of volunteers who had not previously restricted their meat consumption, an important factor to consider in the analysis. To date, data on the beneficial effects of specific types of plant-based diets on body weight and selected metabolic-related parameters have primarily come from studies involving individuals who have been limiting their meat and zoonotic intake for an extended period. Such individuals are also often characterized by higher dietary awareness (76).

The provision of a range of educational materials, along with ready-made diet plans accompanied by detailed instructions for the preparation of recipes, facilitated patients' adherence to the dietary model assigned during the randomisation process. This approach also served to mitigate the risk of potential nutritional deficiencies. Participants in the study were able to provide essential nutrients for plant-based diets, including protein, calcium, iron and iodine. Each group received an equivalent daily dose of vitamin B12. In order to guarantee that the results were consistent across all groups, the participants in each group were required to ingest precisely 100 micrograms of vitamin B12 on a daily basis. Moreover, the implementation of an intensive support system comprising experts from various professional backgrounds, including dieticians, doctors and diagnosticians, in conjunction with the scheduling of frequent meetings, has been instrumental in ensuring the success of the study. This collaborative approach has resulted in a remarkably low dropout rate of only 5.6%. Additionally, the patients were granted the opportunity to pose inquiries to the experts, thereby facilitating the discussion of the challenges encountered during the dietary regimen, which undoubtedly played a pivotal role in determining their commitment to persevering in the VEGPREV study. Nevertheless, it is recommended that patients' motivation be measured during the study using appropriate psychological tools.

A distinguishing feature of the program was the extensive evaluation of diverse plant-based diets. A direct comparison of the various dietary interventions enables the relative effectiveness of the different diets to be evaluated simultaneously, thus saving researchers time. The subjective reactions of the participants during the meetings, in response to the different models, were observed, and it was found that these reactions held great educational value for the authors, who are practicing dietitians and researchers. However, implementing this approach is challenging, necessitating the allocation of additional resources to the recruitment process, the meticulous randomization of participants, and strict adherence to the stipulated protocol by all researchers involved.

The success of this study is also largely attributed to be development of a practical regimen that can be implemented in various dietetic practices. The study participants met with researchers on three occasions over the course of 12 weeks: at the beginning of the study, after 6 weeks and after 12 weeks. These meetings were conducted one-to-one, during which all measurements, blood draws and dietary/nutrition education took place. Between in-person meetings, participants maintained regular communication with a trained psychodietitian and had the opportunity to submit queries via email to the lead dietitian responsible for the educational aspects of the study. Importantly, neither the patients nor the research team faced the significant costs associated with the procedures or the burden of excessive time commitments. In contrast to current practices in other studies, which may prove unrealistic for implementation in dietetic settings (43, 77, 78), this approach offers a more feasible alternative. Additionally, the involvement of a chef and the extensive training required could place a substantial burden on health systems in many countries, potentially extending the time need to achieve measurable results.

## 5 Conclusions

In a 12-week dietary intervention based on four plant-based eating patterns, the most pronounced and consistent changes in body weight, waist circumference, and body fat percentage were observed in the VG and EAT groups. The magnitude of these alterations was greater than in the other intervention groups and did not occur in the control group. No significant changes were observed between the groups in the remaining analyzed parameters, including glycemia, insulinemia, HOMA-IR, blood pressure, lipid profile, appetitive traits, physical activity level, and quality of life. This finding suggests that the impact of plant-based diets in this intervention was predominantly on anthropometric parameters. The EAT and VG diets have been demonstrated to be efficacious tools in the management of weight and body composition in overweight and obese individuals. These findings provide a compelling rationale for the further exploration of sustainable eating patterns as therapeutic strategies and their potential for public health interventions. This will assist in the promotion of more sustainable dietary patterns that reduce carbon footprint and natural resource consumption in the face of the growing popularity of animal-based diets. However, further research in this area is required, including the investigation of larger populations and longer follow-up periods.

## Data Availability

The raw data supporting the conclusions of this article will be made available by the authors, without undue reservation.
